# Inflammatory cytokines in aqueous humor of patients with choroidal neovascularization

**Published:** 2012-03-02

**Authors:** Heng Miao, Yong Tao, Xiao-xin Li

**Affiliations:** 1 Department of Ophthalmology, People’s Hospital, Peking University, Beijing, China; 2 Key Laboratory of Vision Loss and Restoration, Ministry of Education, Beijing, China

## Abstract

**Objective:**

To investigate the correlations between aqueous concentrations of interleukin 1β, 6, 8, 10, 12p (IL-1β, IL-6, IL-8, IL-10, IL-12p), and tumor necrosis factor α (TNF-α) and the parameters of macular edema acquired by optical coherence tomography (OCT) in patients with choroidal neovascularization.

**Methods:**

IL-1β, IL-6, IL-8, IL-10, IL-12p, and TNF-α in the aqueous humor samples of 17 patients with exudative age-related macular degeneration (AMD), ten patients with pathological myopia (PM), seven patients with idiopathic choroidal neovascularization (CNV), and 14 patients with cataract and idiopathic epiretinal membrane or macular hole in the control group were measured with cytometric bead array. The maximum macular thickness and macular volume within 1 mm, 3 mm, and 6 mm were measured with OCT.

**Results:**

In the CNV groups, the aqueous levels of IL-6 and IL-8 were significantly associated with macular volume within 6 mm (p=0.011, p=0.008, respectively), while IL-1β, IL-10, IL-12p, and TNF-α showed no significant correlation with either the maximum macular thickness or the macular volume. By further selecting patients with CNV who had accepted their last intravitreal injection of bevacizumab within 3 months, the level of IL-6 still significantly correlated with the maximum macular thickness (p=0.019) and macular volume within 1 mm (p=0.018), 3 mm (p=0.018), and 6 mm (p=0.022). In patients with exudative AMD, the level of IL-6 was significantly associated with the maximum macular thickness (p=0.025) and macular volume within 1 mm (p=0.025), 3 mm (p=0.006), and 6 mm (p=0.002). The aqueous level of all cytokines did not vary significantly between the CNV patients who had accepted their last intravitreal injection of bevacizumab within 3 months and the other patients, nor was a difference found among patients with exudative AMD, PM, and idiopathic CNV, and the control group.

**Conclusions:**

Intraocular concentrations of IL-6 and IL-8 (particularly IL-6) are significantly associated with the volume of macular edema in patients with CNV. However, intravitreal injection of antivascular endothelial growth factor drugs did not change the intraocular level of these inflammation cytokines.

## Introduction

Choroidal neovascularization (CNV), either idiopathic CNV [1], exudative age-related macular degeneration (AMD) [2], or secondary to pathological myopia (PM) [3], is one of the main causes of vision impairment throughout the world.

Vascular endothelial growth factor (VEGF), first discovered as a vasopermeability factor [4], has been reported to be associated with CNV. Many clinical trials have showed encouraging outcomes for intravitreal injections of anti-VEGF drugs for managing CNV [5-7]. Some authors even recommend anti-VEGF as first-line treatment for some types of CNV [8-10]. However, in addition to anti-VEGF pharmacotherapy, intravitreal administered anti-inflammatory substances, such as triamcinolone acetonide (TA) [11-13], a widely used anti-inflammatory drug, and infliximab [14], an antibody of tumor necrosis factor α (TNF- α), have also shown positive effects in treating CNV in patients and animal models. Therefore, in the era of anti-VEGF, one may postulate that investigating the role of inflammatory factors in the development of CNV has become more prominent.

In this study, we explored the relationship between levels of inflammatory cytokines in aqueous humor of patients with CNV after and not after recent anti-VEGF therapy and the parameters of the macula acquired with optical coherence tomography (OCT).

## Methods

This study included 17 patients (17 eyes) with exudative AMD, ten patients (ten eyes) with pathological myopia (PM), seven patients (seven eyes) with idiopathic choroidal neovascularization (CNV) who underwent intravitreal injection of bevacizumab (Avastin, Genentech Inc., San Francisco, CA) as the study group, and 14 patients (14 eyes) with cataract and idiopathic epiretinal membrane or a macular hole who underwent combined cataract and vitrectomy surgery (CCVS) as the control group. The patients were consecutive in each group. The inclusion criterion in the study group was the presence of active CNV. Exclusion criteria included glaucoma, previous photodynamic therapy, and other retinal diseases, such as diabetic retinopathy and retinal vascular occlusion.

All patients underwent an ophthalmic examination including best-corrected visual acuity (BCVA) recording using manifest refraction and the logMAR visual acuity chart, non-contact tonometry, slit lamp assisted biomicroscopy of the anterior segment and posterior segment of the eye, fundus fluorescein angiography (FFA), and OCT (Optovue OCT-IV, Optovue Inc., Fremont, CA). All patients in the study group showed leakage on fluorescein angiographies (active CNV), and they were further divided into two subgroups according to the interval between the last intravitreal injection of bevacizumab (IVB) and this time (>3 month group, more than 3 months, or <3 month group, less than 3 months). OCT was analyzed by one investigator, and the maximum macular thickness and macular volume within 1 mm, 3 mm, and 6 mm were measured using built-in software. The OCT measurements were converted from millimeters to proportionally corresponding micrometers.

Aqueous humor was collected during intravitreal injection or CCVS. All procedures conformed to the Declaration of Helsinki for research involving human subjects. Informed consent was obtained from all participants. Undiluted aqueous humor samples (100–200 µl) were obtained through anterior chamber paracentesis. All injections and sample collections were performed using a standard sterilization procedure that included the use of topical povidone-iodine and levofloxacin drops. Samples were stored in a sterilized plastic Corning (2 ml, Corning Inc., NY) at −70 °C until use.

The concentration of aqueous interleukin 1β, 6, 8, 10, 12p (IL-1β, IL-6, IL-8, IL-10, IL-12p) and TNF-α were measured with cytometric bead array (CBA), a method for capturing a soluble analyte or set of analytes with beads of known size and fluorescence, making it possible to detect analytes using flow cytometry. Each capture bead has been conjugated with a specified antibody. The detection reagent is a mixture of phycoerythrin (PE)-conjugated antibodies, which provides a fluorescent signal in proportion to the amount of bound analyte. When capture beads and detector reagent are incubated with an unknown sample containing recognized analytes, sandwich complexes (capture bead+analyte+detection reagent) are formed that can be measured using flow cytometry (BD FACSCalibur, BD Bioscience, San Jose, CA) to identify particles with the fluorescence characteristics of the bead and detector. Six bead population with distinct fluorescence intensities coated with specific capture antibodies for IL-1β, IL-6, IL-8, IL-10, IL-12p, and TNF-α were mixed together to form the bead array. Thus, the concentration of each cytokine in one unknown sample can be calculated from regressive analysis of the standard curve simultaneously. In this study, 50 μl of undiluted aqueous humor sample was used for each test. Quality control procedures were performed according to the CBA kit instructions.

The Pearson correlation test was used to explore the relationship between the aqueous level of a cytokine and the OCT parameters (Statistical Package for Social Sciences [SPSS], ver. 17.0; SPSS, Chicago, IL). Parameters were compared using the Mann–Whitney U test between two groups and the Kruskal–Wallis H test to compare variables among various groups. The chi-square test and the Fisher exact *t* test were used to compare noncontinuous variables. Values were reported as mean±standard deviation (SD). Statistical significance was set at p<0.05 for two-tail analysis.

## Results

A total of 48 aqueous humor samples from 48 patients were collected (Table 1). The age of AMD and control group was significantly older than the other two groups (p<0.001), while gender, BCVA and IOP did not significantly vary among groups (all p>0.05).

**Table 1:** Composition of the study population (mean±SD).

** **	**Control**	**Exudative AMD**	**PM**	**Idiopathic CNV**	**p-value**
Number	14	17	10	7	** **
Age (years)	62.4±14.5	68.6±12	49.4±10	33.7±10.4	<0.001*
Female-no (%)	11 (79%)	5 (29.4%)	5(50%)	4 (57%)	0.057**
BCVA	0.2±0.13	0.24±0.19	0.20±0.23	0.36±0.36	0.787*
IOP (mmHg)	14.3±2.6	13.6±3.0	12.3±2.6	14.86±3.08	0.313*

*Kruskal–Wallis H test, compared among control group, exudative AMD, PM and idiopathic CNV groups. **Fisher’s exact *t*-test, compared among control group, exudative AMD, PM and idiopathic CNV groups. AMD, age-related macular degeneration; PM, pathological myopia; CNV, choroidal neovascularization; BCVA, best corrected visual acuity; IOP, intraocular pressure.

**Figure 1:**
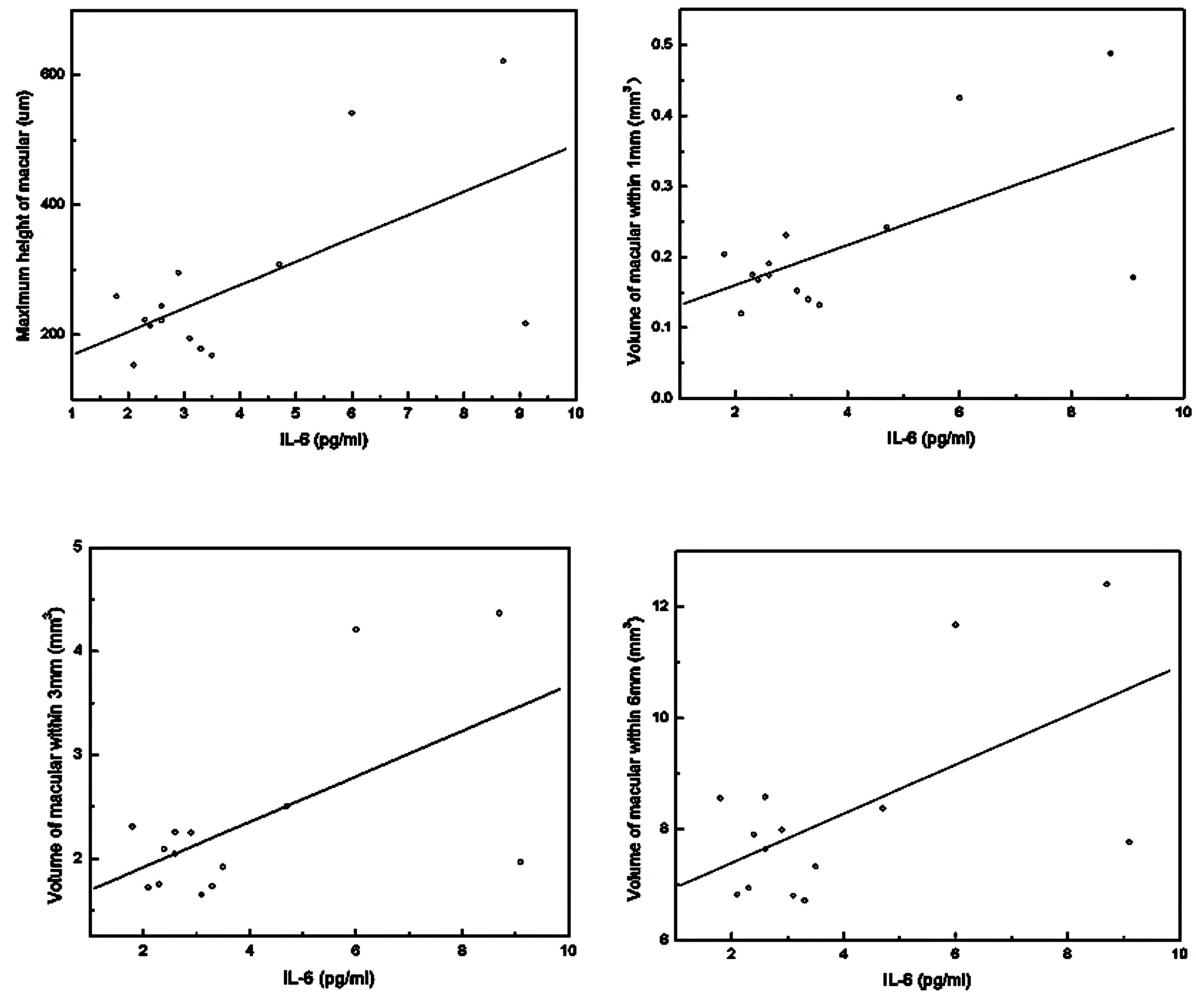
Interleukin (IL)-6 associated significantly with maximum macular thickness (p=0.019, R^2^=0.381), volume of macula within 1 mm (p=0.018, R^2^=0.382), 3 mm (p=0.018, R^2^=0.382) and 6 mm (p=0.022, R^2^=0.366) in choroidal neovascularization (CNV) group who accepted IVB within 3 months.

In the CNV group, the aqueous levels of IL-6 and IL-8 were significantly associated with macular volume within 6 mm (P_IL-6_=0.011, R^2^=0.176, P_IL-8_=0.008, R^2^=0.187; Table 2), while IL-1β, IL-10, IL-12p, and TNF-α showed no significant correlation with the maximum macular thickness or macular volume within 1 mm, 3 mm, or 6 mm (all p>0.05). Moreover, in the CNV group who had accepted their last IVB within the last 3 months (<3 months), the level of IL-6 correlated significantly with the maximum macular thickness and macular volume within 1 mm (p=0.019, R^2^=0.382), 3 mm (p=0.018, R^2^=0.382), and 6 mm (p=0.022, R^2^=0.366; Table 2, Figure 1). However, in the CNV group who had accepted their last IVB more than 3 months before or never (>3 months), no significant correlation was found between the cytokine concentrations and the macular parameters (all p>0.05).

**Table 2:** P values of each Pearson correlation test between level of cytokines and OCT parameters in CNV groups.

	**CNV**				**<3 mon**				**>3 mon**			
**Cytokines**	**MMT**	**MV** **(1 mm)**	**MV** **(3 mm)**	**MV** **(6 mm)**	**MMT**	**MV** **(1 mm)**	**MV** **(3 mm)**	**MV** **(6 mm)**	**MMT**	**MV** **(1 mm)**	**MV** **(3 mm)**	**MV** **(6 mm)**
IL-1b	0.614	0.134	0.331	0.186	0.951	0.950	0.981	0.851	0.373	0.071	0.057	0.052
IL-6	0.063	0.393	0.094	0.011*	0.019*	0.018*	0.018*	0.022*	0.640	0.989	0.809	0.549
IL-8	0.074	0.212	0.081	0.008*	0.517	0.515	0.950	0.901	0.195	0.870	0.219	0.066
IL-10	0.602	0.816	0.399	0.120	0.117	0.119	0.184	0.079	0.350	0.628	0.727	0.935
IL-12p	0.851	0.443	0.936	0.898	0.487	0.487	0.591	0.519	0.924	0.172	0.700	0.631
TNF-α	0.051	0.185	0.309	0.480	0.225	0.225	0.434	0.560	0.150	0.437	0.503	0.644

CNV, choroidal neovascularization group; <3mon, patients who receive last IVB within 3 months; >3mon, patients who receive last IVB for more than 3 months and never; MMT, maximum macular thickness; MV, volume of macula; IL, interleukin; TNF, tumor necrosis factor.

In exudative AMD (Table 3), the level of IL-6 was also significantly associated with the maximum macular thickness (p=0.025, R^2^=0.258) and macular volume within 1 mm (p=0.025, R^2^=0.258), 3 mm (p=0.006, R^2^=0.386), and 6 mm (p=0.002, R^2^=0.461).

**Table 3:** P values of each Pearson correlation test between level of cytokines and OCT parameters in exudative AMD.

**Cytokines**	**MMT**	**MV (1 mm)**	**MV (3 mm)**	**MV (6 mm)**
IL-1b	0.730	0.730	0.888	0.922
IL-6	0.025*	0.025*	0.006*	0.002*
IL-8	0.150	0.152	0.110	0.087
IL-10	0.306	0.309	0.174	0.092
IL-12p	0.535	0.534	0.588	0.408
TNF-α	0.123	0.122	0.2414	0.128

MMT, maximum macular thickness; MV, volume of macula; IL, interleukin; TNF, tumor necrosis factor.

The aqueous level of all cytokines investigated in this study showed no significant difference between the patients with CNV who had accepted their last IVB within the last 3 months (<3 months), patients with CNV who had accepted their last IVB more than 3 months before or never (>3 months), and the control group (all p>0.05, Table 4). Moreover, no difference was found among the patients with exudative AMD, PM, and idiopathic CNV compared to the control group, either (all p>0.05, Table 4).

**Table 4:** Levels of cytokines in CNV and control group (mean±SD).

**Cytokines**	**<3 mon**	**>3 mon**	**Control**	P*	**Exudative AMD**	**PM**	**Idiopathic CNV**	p*
IL-1b	1.98±0.28	1.99±0.23	1.94±0.20	0.672	2.1±0.3	1.9±0.2	2.0±0.2	0.287
IL-6	3.94±2.37	4.49±3.09	4.47±2.07	0.610	3.6±1.8	4.9±2.0	3.1±1.5	0.124
IL-8	4.18±1.22	5.52±2.59	5.51±2.90	0.118	4.5±1.5	4.9±0.8	4.3±1.0	0.145
IL-10	1.82±0.23	1.85±0.25	1.90±0.17	0.257	1.8±0.3	1.9±0.1	1.8±0.3	0.258
IL-12p	2.08±0.19	2.19±0.31	2.19±0.27	0.444	2.2±0.3	2.2±0.2	2.1±0.3	0.755
TNF-α	1.90±0.22	1.85±0.27	1.95±0.28	0.507	1.9±0.2	1.8±0.2	2.0±0.4	0.621

*Kruskal–Wallis H test was used. CNV, choroidal neovascularization group; <3mon, patients who receive last IVB within 3 months; >3mon, patients who receive last IVB for more than 3 months and never; Control, patients with cataract and idiopathic epiretinal membrane or macular hole who underwent combined cataract and vitrectomy surgery; AMD, age-related macular degeneration; PM, pathological myopia; CNV, choroidal neovascularization; IL, interleukin; TNF, tumor necrosis factor.

## Discussion

Although CNV is not a classic inflammatory disease like uveitis, much evidence from anatomic studies [15,16] and molecular experiments [17-19] has supported the hypothesis that inflammation plays an important role in disease pathogenesis and progression. Innate immunity and autoimmune components are believed to be heavily involved in CNV development. Macrophage, lymphocytes, and neutrophils have been found to be important in animal models and CNV patients [20-22]. IL-6 is a cytokine derived from activated T lymphocytes with multiple functions, including induction of B-cell growth, induction of B-cell differentiation and antibody production, induction of differentiation and proliferation of T cells, synergistic induction with IL-3 of hematopoietic cell growth, and induction of hepatocyte secretion of acute-phase inflammatory proteins [23,24]. IL-8 is a group of peptides produced by various types of cell, which activate and recruit polymorphonuclear leukocytes in acute and chronic inflammatory process, and are probably involved in initiating labor and delivery in pregnant women [25,26]. Both were found elevated in the synovial fluid of patients who had rheumatoid arthritis. This evidence suggested the role of cytokines in inflammatory diseases [27].

Our results indicated that the aqueous level of IL-6 and IL-8 was significantly associated with the macular volume within 6 mm in patients with CNV, and the aqueous level of IL-6 was significantly associated with the maximum macular thickness and macular volume within 1 mm, 3 mm, and 6 mm in patients who had received IVB within the last 3 months. This implied that IL-6 and IL-8, especially IL-6, not only participate in the process of CNV but also may be another target molecule in treating idiopathic CNV, exudative AMD, and CNV secondary to PM. Furthermore, a macular volume within 6 mm could also be an indicator of the aqueous level of these cytokines in the follow-up process, especially for patients who had accepted IVB within the last 3 months. We also found that the concentration of IL-6 had a linear correlation with the maximum height of macula and macular volume within 1 mm, 3 mm, and 6 mm in exudative AMD. Thus, anti-IL-6 therapy may be further considered in this anti-VEGF era.

Our results also suggested that the aqueous level of cytokines investigated in this study did not significantly vary between patients with CNV who had undergone IVB within the last 3 months and the other patients with CNV. This may explain why some patients were resistant to anti-VEGF therapy. IVB could downregulate only the VEGF concentration, not other cytokines such as IL-6 and IL-8. Anti-inflammatory agents may still be necessary for those patients after failed anti-VEGF therapy.

We could not find any significant correlation between the concentrations of IL-1β, IL-10, IL-12p, and TNF-α and any OCT parameters in any group in this study, which suggested that their role in the progress of CNV may be not as pivotal as that of anti-IL-6 and anti-IL-8.

Our results were in agreement with several previous studies. Higgins [28] found that the response to reactive oxygen species in the photoreceptor outer segments of inflammatory injury, commonly believed to be an initiation of AMD, could lead to increased expression and secretion of IL-8. Goverdhan [29] also suggested that the −251A allele (rs4073) of the IL-8 promoter is more prevalent in AMD cases than controls. Izumi-Nagai et al. [30] found that the IL-6 receptor mediated activation of the signal transducer and activator of transcription-3 promotes CNV generation and the IL-6 receptor blockade could inhibit in vivo and in vitro expression of inflammation-related molecules such as monocyte chemotactic protein, intercellular adhesion molecule-1, VEGF, and macrophage infiltration, which then could be a therapeutic strategy to suppress CNV-associated AMD.

There were limitations in this study. First, the selection of the control group may have caused selection bias, since the aqueous level of all cytokines investigated in this study did not vary significantly between the CNV group and the control group (all p>0.05). Patients with idiopathic epiretinal membrane or a macular hole [31-35] were the preferred control in most previous cytokine research of fundus diseases. However, a slight inflammation process may also potentially be involved in the formation of idiopathic epiretinal membrane or a macular hole; therefore, we have to acknowledge that the aqueous level of all the cytokines investigated in this study of patients with CNV may possibly differ from that of normal cataract patients without epiretinal membrane or a macular hole. Further study will be conducted to evaluate this issue. Second, the number of enrolled patients was not large, which may cover some minor significant association between factors. With a larger number of study participants, the correlations between the aqueous concentrations of the cytokines and the macular thickness or macular volume may become statistically significant. Third, only six cytokines were detected in this study, which fail to represent the main pathological mechanism involved in the development of CNV. Fourth, the aqueous humor samples may be not as valuable as vitreous fluid for detecting cytokine concentrations for fundus diseases. However, obtaining vitreous samples needs to expand the surgical intervention while CNV was not the surgical indication of vitrectomy for most cases.

A previous study investigated the relationship of recurrent CNV and cytokines, but this study may be the first one that states the association between cytokines and CNV volume, suggesting their importance. In conclusion, the aqueous level of IL-6 is positively related to the maximum macular thickness and macular volume within 1 mm, 3 mm, and 6 mm in patients with CNV who had undergone their last IVB within the last 3 months. Intravitreal injection of anti-VEGF drugs did not change the intraocular level of inflammation cytokines, including IL-1β, IL-6, IL-8, IL-10, IL-12p, and TNF-α. Furthermore, IL-6 is positively related to these OCT parameters in exudative AMD. IL-6 and IL-8, especially the former, may be additional target molecules in therapy for CNV.
